# Genome Sequence of a *Pseudomonas syringae* pv. tabaci Strain, yuexi-1, Causing Wildfire Disease in Tobacco

**DOI:** 10.1128/genomeA.00180-15

**Published:** 2015-04-09

**Authors:** Tielin Wang, Yuwen Yang, Tingchang Zhao

**Affiliations:** State Key Laboratory for Biology of Plant Diseases and Insect Pests, Institute of Plant Protection, Chinese Academy of Agricultural Sciences, Beijing, China

## Abstract

We determined the draft genome sequence of the *Pseudomonas syringae* pv. tabaci strain yuexi-1. It was isolated from tobacco sample of yuexi-1, Sichuan province, China, by our laboratory. The genome contains 6,232,497 bp and has a G+C content of 58.2 mol%.

## GENOME ANNOUNCEMENT

Tobacco wildfire disease caused by *Pseudomonas syringae* pv. tabaci is a kind of bacterial leaf disease. It occurs during the seedling stage and field phase, which does harm mainly to blades and also to young stems, capsules, sepals, and so on (1). However, it is difficult to select resistant cultivars due to the great genetic diversity of *Pseudomonas syringae* pv. tabaci (2). Two strains, *Pseudomonas syringae* pv. tabaci 6605 and *Pseudomonas syringae* pv. tabaci ATCC 11528, have been submitted by the University of Exeter and the University of North Carolina at Chapel Hill, respectively (3, 4). In this paper, we report the complete genomic sequence of *Pseudomonas syringae* pv. tabaci strain yuexi-1. It would be helpful for understanding the genetics and pathogenic mechanism of *Pseudomonas syringae* pv. tabaci through comparative genomics. The *Pseudomonas syringae* pv. tabaci strain yuexi-1, which was isolated from a tobacco sample from Yuexi, Sichuan Province, China, which had a high virulence to the host. Genomic DNA of *Pseudomonas syringae* pv. tabaci strain yuexi-1was extracted from a triply cloned pure culture in King’s medium B using a genome extraction kit (BioTeke, Beijing, China) according to the manufacturer’s instructions. The genome of the *Pseudomonas syringae* pv. tabaci strain yuexi-1 was sequenced with massively parallel sequencing (MPS) Illumina technology. Library construction and sequencing were performed at Sangon Biotech (Shanghai), China. Two DNA libraries were constructed, a paired-end library with an insert size of 300 to 400 bp and a mate-pair library with an insert size of 5 kb. Using Velvet version 1.2.07, we assembled the genome into a total length of 6,232,497 bases and 106 scaffolds. The largest scaffold was 1,168,964 bases. Among the large scaffolds, the *N*_50_ size was 573,229 bases. The scaffolds had an average length of 204,310 bases. Gene prediction was performed on the *Pseudomonas syringae* pv. tabaci strain ATCC 11528 genome. A whole-genome Blast search (E value, ≤1e^−5^; minimal alignment length percentage, ≥40%) was performed against 5 databases: the Kyoto Encyclopedia of Genes and Genomes (KEGG) (5), the NCBI nonredundant protein database (NR) (6), Swiss-Prot (7), Clusters of Orthologous Groups (COG) (8), and Gene Ontology (GO) (9).

The genome contains a single circular chromosome of 6,232,497 bp with a GC content of 58.2 mol%. Total coding genes are 5,354,916 bp, and 5,701 protein-coding genes were identified in the genome, with an average length of 939 bp. A total of 3,261 (57.2%) genes were classified into Clusters of Orthologous Groups (COG) families comprising 21 functional categories.

### Nucleotide sequence accession numbers.

This whole-genome shotgun project has been deposited at DDBJ/EMBL/GenBank under the accession number JWJF00000000. The version described in this paper is version JWJF01000000.
